# Artemether Attenuates High Glucose-Induced Inflammation and Fibrogenesis in Renal Tubular Epithelial Cells by Modulating the TGF-*β*/Smad Pathway Via PPAR*γ* Activation

**DOI:** 10.1155/jdr/5052561

**Published:** 2025-11-29

**Authors:** Xiuli Zhang, Lingzhi Li, Rui Xue, Dan Liang, Yue Wang, Weidong Yang, Qinghui Zhou, Zhihong Chi

**Affiliations:** ^1^Department of Nephrology, The First Affiliated Hospital of Shenzhen University, Shenzhen Second People's Hospital, Shenzhen, Guangdong Province, China; ^2^Rehabilitation Medicine Department, The 964 hospital of PLA, Changchun, Jilin Province, China; ^3^Department of Tissue Culture, Liaoning University of Traditional Chinese Medicine, Shenyang, Liaoning Province, China; ^4^Department of Pathophysiology, China Medical University, Shenyang, Liaoning Province, China

**Keywords:** artemether, diabetic kidney disease, fibrosis, inflammation, TGF-*β*/Smad signaling pathway

## Abstract

Artemether (Art) is a derivative of artemisinin, originally sourced from traditional Chinese herbal medicine, with improved bioavailability, and is widely used for malaria treatment. Recently, its potential effects on diabetic complications, particularly diabetic kidney disease (DKD), have attracted increasing attention. This study aimed to evaluate the therapeutic effects of Art on DKD and to explore the underlying mechanisms. Specifically, we investigated the protective role of Art against high glucose (HG)-induced inflammatory biomarkers (transforming growth factor-*β*1 [TGF-*β*1], tumor necrosis factor-*α* [TNF-*α*], interleukin-1*β* [IL-1*β*], and interleukin-6 [IL-6]) and fibrosis in human renal proximal tubular epithelial (HK-2) cells, focusing on the involvement of peroxisome proliferator-activated receptor gamma (PPAR*γ*) in DKD. To mimic diabetic conditions, HK-2 cells were exposed to HG (30 mM) to induce inflammation and fibrosis. The therapeutic effects of Art (100 and 200 *μ*m) were assessed by immunofluorescence, real-time RT-PCR, and Western blot analyses. Our results demonstrated that Art effectively reversed the HG-induced upregulation of inflammatory and fibrogenic markers in HK-2 cells (∗*p* < 0.05, ∗∗*p* < 0.001, and ⁣^##^*p* < 0.001). Additionally, Art pretreatment restored the HG-suppressed expression of PPAR*γ* (∗*p* < 0.05 and ⁣^##^*p* < 0.001), suggesting that Art exerts its antifibrotic effects by modulating PPAR*γ* and inhibiting the TGF-*β*/Smad pathway. This hypothesis was further supported by siRNA-mediated knockdown of PPAR*γ*, which significantly diminished Art's antifibrotic effects (∗*p* < 0.05, ∗∗*p* < 0.001, and ⁣^##^*p* < 0.001). In conclusion, our study indicates that Art protects renal tubular epithelial cells by partially modulating PPAR*γ*-dependent inhibition of the TGF-*β*/Smad pathway, thereby mitigating HG-induced inflammation and fibrosis. These findings suggest that Art holds promising therapeutic potential for DKD treatment.

## 1. Introduction

Type 2 diabetes mellitus (T2DM) has emerged as one of the most prevalent chronic diseases worldwide, with its incidence rising rapidly in recent years [1]. Diabetic kidney disease (DKD, formerly referred to as diabetic nephropathy, DN) represents one of the most serious microvascular complications of T2DM and is a leading cause of end-stage kidney disease (ESKD), often necessitating renal replacement therapies such as dialysis or kidney transplantation [2]. Consequently, DKD imposes a significant global health burden. The progressive phase of DKD is typically associated with tubulointerstitial fibrosis (TIF), driven by increased expression of profibrotic cytokines such as TGF-*β*1, resulting in progressive loss of tissue structure and renal function [3]. Compelling evidence highlights inflammation as a central contributor to DKD development and progression [4, 5]. Therefore, targeting inflammation and extracellular matrix (ECM) accumulation represents a potential therapeutic strategy for DKD.

Traditional Chinese medicine has been employed in the management of diabetes for centuries. A natural antimalarial drug derived from the Chinese herb *Artemisia annua* (*A. annua*) has already been used in clinic [6–10]. Following advances in research, the effects of artemisinin and its derivatives on metabolic diseases have become an increasing focus of interest in recent years, with an emphasis on their effects on diabetic complications such as DKD [8]. Art (artemether) treatment improves islet function and metabolic homeostasis in diabetic nonhuman primates. In 2019, Han et al. reported that Art reduced urinary albumin excretion and attenuated both glomerular and tubular basement membrane thickening in db/db mice with Type 2 diabetic kidney disease [11]. Similarly, Wang et al. demonstrated the renoprotective effects of Art in a Type 1 diabetes mellitus (T1DM) model, attributing these benefits to improved mitochondrial function and regulation of redox homeostasis in the kidney [12]. Despite these promising findings, the role of Art in diabetes-induced TIF remains poorly understood. Therefore, further investigation is warranted to elucidate the potential mechanisms through which Art may regulate TIF in the context of diabetic kidney injury.

Peroxisome proliferator-activated receptor gamma (PPAR*γ*), a nuclear hormone receptor superfamily member, regulates multiple physiological processes including metabolism, inflammation, and fibrosis [13, 14]. It is abundantly expressed in the glomerulus, proximal tubules, and medullary collecting duct, and is critical for normal renal function [15]. Dysregulation of PPAR*γ* contributes to the pathogenesis of various renal diseases, potentially via modulation of inflammation and mitochondrial function [16, 17]. PPAR*γ* agonists effectively control serum glucose levels in patients with diabetes [18], but some have been withdrawn because of adverse effects such as fluid retention, weight gain, and increased cardiovascular risk [19]. Thus, identifying natural, safer PPAR*γ* agonists is crucial to develop novel therapeutic strategies for DKD, particularly those with anti-inflammatory and antifibrotic properties.

The TGF-*β*/Smad signaling pathway has been extensively studied in renal fibrosis [3, 20]. TGF-*β* induces fibroblast proliferation and ECM accumulation by binding to Type I and II receptors, activating TGF-*β* type I kinase, which phosphorylates Smad2 and Smad3 [21]. Activated Smad2/3 form complexes with Smad4, translocate into the nucleus, and induce gene expression promoting ECM production [22]. Previous studies demonstrate that PPAR-*γ* activation suppresses fibrosis and inflammation by inhibiting TGF-*β*1/Smad signaling in various tissues [23–25]. For instance, El-Kashef et al. reported that Saroglitazar attenuated unilateral ureteral obstruction (UUO)-induced TIF via regulation of TGF-*β*1/Smad3 signaling [23]. Jing Hua et al. found that modulation of PPAR-*γ* affected hepatic stellate cell (HSC) activation through the TGF-*β*1/Smad pathway, influencing liver fibrosis [24]. Recently, Rane et al. showed that PPAR*γ* stimulation combined with TGF-*β*1/Smad3 inhibition suppressed adipose tissue fibrosis and improved metabolism [25]. These findings highlight PPAR*γ* upregulation-mediated inhibition of TGF-*β*/Smad signaling as a key mechanism in fibrosis suppression. However, the antifibrotic capacity of Art and its mechanisms in DKD require further elucidation. Here, we provide evidence that Art alleviates TIF by activating PPAR*γ*-mediated inhibition of the TGF-*β*/Smad pathway, supporting Art as a promising therapeutic strategy for DKD and related fibrosis.

## 2. Material and Methods

### 2.1. Cell Culture

Human renal proximal tubule (HK-2) cell line (Shanghai Cell Bank of Chinese Academy of Sciences) was commercially available. Cells were grown in Dulbecco's modified Eagle's medium (DMEM)/Ham's F12 medium (DMEM/F12) (Invitrogen) supplemented with 10% fetal bovine serum (Invitrogen), and maintained at 37°C in a humidified atmosphere with 5% CO_2_. When the cultured cells were almost confluent state, they were transferred to serum-free DMEM/F12 for 24 h to arrest and synchronize cell growth. The concentration of HG used in the current study was previously described by our group [26]. In the control groups, the cells were exposed only to normal medium. For the other experimental groups, the cells were pretreated with Art (100 or 200 *μ*M; #A9361, St Louis, MO) for 24 h, followed by addition 48-h incubation with 30 mM HG.

### 2.2. Cell Viability Assay

Quantitative colorimetric assay with MTT (3-(4,5-dimethylthiazol-2-yl)-2,5-diphenyltetrazolium bromide) was used for measuring the cell viability. The HK-2 cells grown in 96-well plates were cultured with a density of 5 × 10^4^. Art treatments with the amount of 50, 100, 200, and 300 *μ*M were performed for 24 h, while 10 *μ*L MTT (final concentration, 500 *μ*g/mL) was added to the medium. The Art treated cells were incubated at 37°C for 3 h. Formazan crystals formed in viable cells were dissolved in dimethyl sulfoxide (DMSO; supplier unspecified) and absorbance was subsequently read at 540 nm using a microplate reader. The results were shown as a percentage of MTT reduction in treated cells relative to 100% MTT reduction in the control cells.

### 2.3. Transient Transfection With PPAR*γ*-Small Interfering RNA (siRNA)

HK-2 cells were seeded at a density of 2 × 10^5^ cells/well in 6-well plates containing 2 mL DMEM/F12. Upon reaching 70% confluence, cells were then transfected with PPAR*γ*-specific siRNA (sense, 5′ CCAAGUUUGAGUUGCUGUdTdT 3′ and antisense, 5′ ACAGCAAACUCAAACUU GGdTdT 3′, Santa Cruz Biotechnology, Inc.). To ensure that the target sequences of the siRNA were correctly aligned, BLAST was used on the GenBank database (http://blast.ncbi.nlm.nih.gov/Blast.cgi). The knockdown effect of PPAR*γ* was evaluated by Western blot and RT-PCR at 24, 48, 72, and 96 h post-transfection.

### 2.4. Immunofluorescence Staining

HK-2 cells were fixed with 4% paraformaldehyde and permeabilized using 0.1% Triton X-100. Subsequently, the cells were incubated with primary mouse monoclonal antibodies against PPAR*γ* (Abcam, ab178866, 1:200), Snail (Abcam, ab31787, 1:200), *α*-SMA (Abcam, ab32284, 1:200), p-Smad2 (Invitrogen, 44-244G, 1:200), and p-Smad3 (Invitrogen, 44-246G, 1:200). After three rinses with PBS, the cells were incubated with fluorescent secondary antibodies DAR-FITC (1:50) and Texas Red-DAM (1:50) at room temperature for 2 h, followed by the addition of DAPI nuclear staining at 4°C for 2 h. Finally, the sections were mounted with an anti-fading mounting medium and examined using a confocal laser scanning microscope (SP2, Leica, Germany). Excitation filters for FITC (488 nm) and Texas-Red (568 nm) were used. Images were processed using an Adobe Photoshop program.

### 2.5. Enzyme-Linked Immunosorbent Assay

Levels of transforming growth factor-*β*1 (TGF-*β*1), tumor necrosis factor-*α* (TNF-*α*), interleukin-1*β* (IL-1*β*), and interleukin 6 (IL-6) in HK-2 cell were estimated by ELISA kits in according to manufacturer's recommendations.

### 2.6. Real-Time RT-PCR

Total RNA was extracted from cultured HK-2 cells using TRIzol reagent (Invitrogen). The sequences of the PPAR-*γ* gene were obtained from the GenBank database, and specific primers (Fw: 5′-CTGCATGTGATCAAGAAGAC-3′ and Rv: 5′-AGTGC AATCAATAGAAGGAAC-3′) were designed using Primer Premier 5.0 and synthesized by Takara Biotechnology. PCR amplification and detection were performed using Light Cycler 480 (Roche, Basel, Switzerland). The relative mRNA levels of the target genes were normalized to the expression of GAPDH using the simplified comparative threshold cycle. Subsequently, the processed data were analyzed using Advanced Relative Quantification software (Roche).

### 2.7. Western Blot Analysis

Proteins were extracted from HK-2 cells, and the concentration was determined using the Bio-Rad Protein Assay Kit (Bio Rad Laboratories, Inc., Hercules, CA, United States) [26]. Subsequently, 20 *μ*g of the proteins were loaded and run on SDS-PAGE, and then transferred to PVDF membranes (Millipore, Temecula, CA, United States). The membranes were then incubated overnight with a variety of primary antibodies, including Anti-TGF*β*-1 (Abcam, ab215715, 1:800), Anti-Smad2 (Abcam, ab40855, 1:800), Anti-p-Smad2 (Abcam, ab2800888, 1:600), Anti-Smad3 (Abcam, ab408541:600), Anti-p-Smad3 (Abcam, ab52903, 1:600), Anti-snail (Abcam, ab216347,1:800), Anti-*α*-SMA (Sigma-Aldrich, St. Louis, MO, United States,1:800), Anti-collagen-I (Abcam, ab138492, 1:800), Anti-PPAR*γ* (Abcam, ab178860,1:800), Anti-pPPAR*γ* (LS-C416660 LifeSpan BioSciences, 1:800), and mouse monoclonal anti-GAPDH (#5174 Cell Signaling Technology, 1:2000). Accordingly, appropriate secondary antibodies, such as rabbit anti-goat IgG and rabbit anti-mouse IgG, were incubated at 4°C. The membrane was washed with TBST buffer three times for 10 min each. The membranes were incubated in ECL (Pierce, Thermo Co. Ltd., United States) reagent for HRP (30 s) and exposure to autoradiography film for visualization of the bands. The relative amounts of various proteins were analyzed. The results were quantified by Quantity One Software. Quantitative analysis was performed by measuring the fluorescence intensity compared with the control. Data were expressed as mean ± SEM of at least three experiments.

## 3. Statistical Analysis

Data were presented as mean ± SEM and analyzed with SPSS version 18 (IBM Corporation, Armonk, NY, United States). All experiments were performed at least three times, and their variance was evaluated using the standard ANOVA, with significance determined by one-way ANOVA and Tukey's multiple comparison tests for comparisons between groups. Results were considered significant as *p* < 0.05.

## 4. Results

### 4.1. Art Inhibits HG-Induced Fibrogenesis in HK-2 Cells

**Figure 1:**
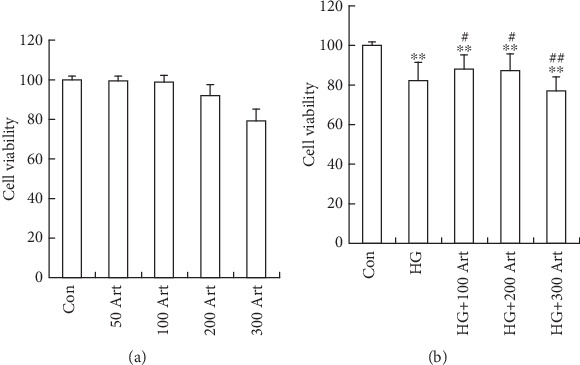
Effects of Art on the cell viability. (a) The HK-2 cells were treated with various doses of Art (50, 100, 200, or 300 *μ*M), the cell viability was analyzed by MTT assay. (b) The HK-2 cells were treated with Art 100, 200,or 300 *μ*M for 24 h followed HG 48 h, and the cell viability was analyzed by MTT assay. The data were mean ± SEM. ⁣^∗^*p* < 0.05 and ⁣^∗∗^*p* < 0.001 vs. Control ⁣^∗^*p* < 0.05 and ⁣^##^*p* < 0.001 vs. HG.

**Figure 2:**
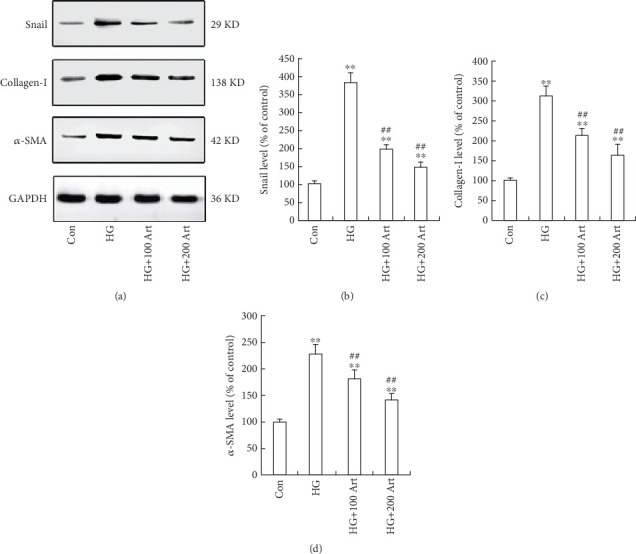
Art inhibits HG-induced fibrogenesis in HK-2 cells. Western blot showing fibrogenesis expression in the cells fractions of HK-2 cells treated with HG (30 mM, 48 h), (a-d). ⁣^∗^*p* < 0.05 and ⁣^∗∗^*p* < 0.001 vs. Control ⁣^#^*p* < 0.05 and ⁣^##^*p* < 0.001 vs. HG.

**Figure 3:**
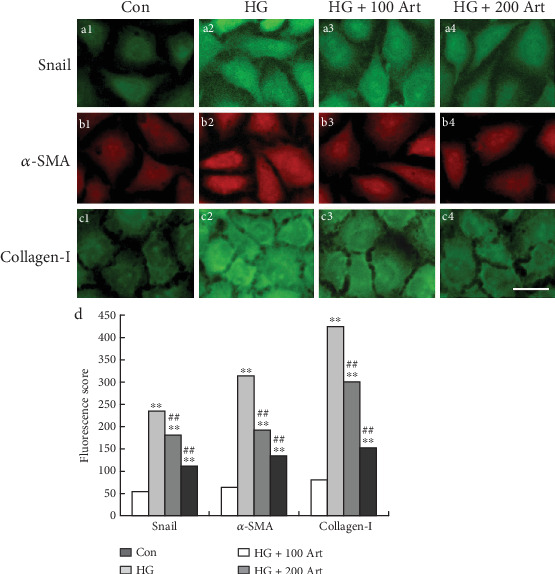
Art inhibits HG-induced fibrogenesis in HK-2 cells. Panels (a–c) show the fibrogenesis proteins expression which assessed by using immunofluorescence staining. a1, b1, c1 the control group; a2, b2, c2, the HG group; a3, b3, c3, HG + 100 Art group; a4, b4, c4, HG + 200 Art group. Results are representative of three experiments (bar 10 *μ*m). (d) The data were mean ± SEM). ⁣^∗^*p* < 0.05 and ⁣^∗∗^*p* < 0.001 vs. Control. ⁣^∗^*p* < 0.05 and ⁣^##^*p* < 0.001 vs. HG.

The effect of Art on HG-induced fibrogenesis in HK-2 cells was investigated. First, to avoid the influence of cell toxicity in the following experiments, we performed the cell viability assay to select optimal concentrations, as previously reported [12, 27–28]. Accordingly, 100 and 200 *μ*M Art were chosen as optimal concentrations in the present study (Figure 1a,b, ⁣^∗^*p* < 0.05 and ⁣^∗∗^*p* < 0.001 vs. Control; ⁣^∗^*p* < 0.05 and ⁣^##^*p* < 0.001 vs. HG). To further investigate HG-induced fibrogenesis, we assessed the levels of snail, *α*-SMA, and collagen-I by Western blot in the presence or absence of Art under HG conditions. As shown in Figures 2a, 2b, 2c, and 2d, the protein expressions of these fibrogenesis markers were upregulated, confirming that HG promotes fibrosis in HK-2 cells. Remarkably, pretreatment of the HK-2 cells with Art attenuated the HG-induced upregulation of fibrogenesis. This inhibitory effect of Art was further confirmed by immunofluorescence staining. HG treatment of the cells for 48 h led to cell morphological change to a fibroblast-like shape, accompanied by increased expression of snail, *α*-SMA, and collagen-I, which were all attenuated by pretreatment with 100 or 200 *μ*M Art (Figures 3a, 3b, and 3c, ⁣^∗^*p* < 0.05 and ⁣^∗∗^*p* < 0.001 vs. Control; ⁣^∗^*p* < 0.05 and ⁣^##^*p* < 0.001 vs. HG), consistent with the results of Western blot analysis. These data suggest that Art inhibits HG-induced fibrosis in HK-2 cells.

### 4.2. Art Inhibits HG-Induced Inflammatory Response in HK-2 Cells

**Figure 4:**
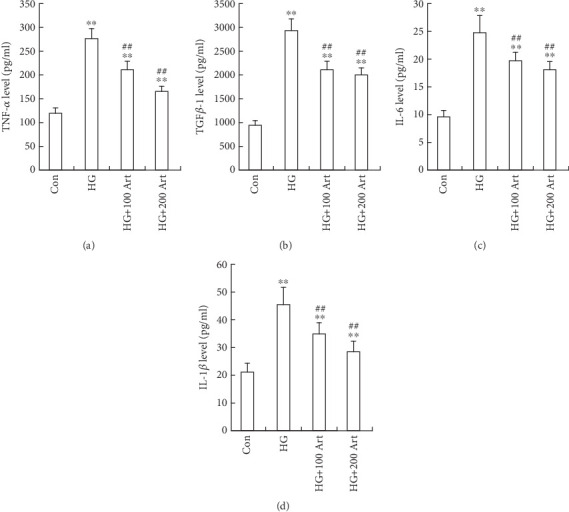
Art inhibits HG-induced inflammatory response in HK-2 cells ELISA showing inflammatory factor (a) TNF-*α*, (b) TGF-*β*1, (c) IL-6 and (d) IL-1*β* expressions in the HK-2 cells treated with HG (30 mM, 48 h) with or without Art. The data were mean ± SEM. ⁣^∗^*p* < 0.05 and ⁣^∗∗^*p* < 0.001 vs. Control. ⁣^#^*p* < 0.05 and ⁣^##^*p* < 0.001 vs. HG.

HG stimulation markedly elevated the expression levels of key pro-inflammatory cytokines, including TNF-*α*, TGF-*β*1, IL-6, and IL-1*β* in HK-2 cells, which is consistent with inflammation-driven pathogenesis in DKD. Notably, treatment with Art significantly suppressed these HG-induced increases (Figures 4a, 4b, 4c, and 4d, ⁣^∗^*p* < 0.05 and ⁣^∗∗^*p* < 0.001 vs. Control; ⁣^#^*p* < 0.05 and ⁣^##^*p* < 0.001 vs. HG), indicating a potent anti-inflammatory effect. These findings suggest that Art may exert renal protective effects by downregulating pro-inflammatory mediators, thereby potentially interrupting the inflammatory cascade that contributes to tubulointerstitial damage in DKD.

### 4.3. Influence of Art on HG-Induced PPAR*γ* in HK-2 Cells

**Figure 5:**
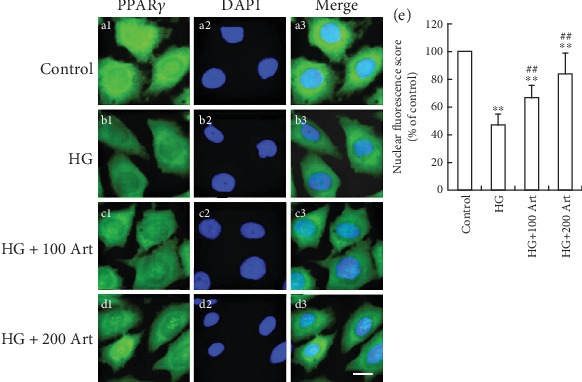
Influence of Art on HG-induced PPAR*γ* in HK-2 cells by immunofluorescence staining analysis. The HK-2 cells were pretreated with artemether 100 or 200 *μ*M for 24 h before exposure to 30 mM HG for 48 h. The PPAR*γ* expression was assessed by immunofluorescence staining analysis as described in the Materials and Methods section (a1 through d3). (a1–a3) the control group; (b1–b3), the HG group; (c1–c3) HG + 100 Art group; (d1–d3) HG + 200 Art group. Results are representative of three experiments (bar 5 *μ*m) (Magnification, ×200). (e) Relative PPAR*γ* fluorescaping score in HK-2 cells. ⁣^∗^*P* < 0.05 and ⁣^∗∗^*P* < 0.001 vs. Control. ⁣^#^*P* < 0.05 and ⁣^##^*P* < 0.001 vs. HG.

**Figure 6:**
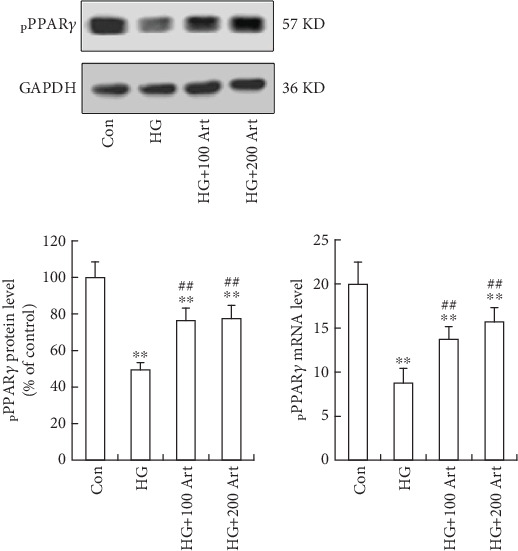
Influence of Art on HG-induced PPAR*γ* expression in HK-2 cells pPPAR-*γ* expression of gene and protein levels by RT-PCR and Western blot analysis. The HK-2cells were pretreated with artemether 100 or 200 *μ*M for 24 h before exposure to 30 mM HG for 48 h. The data were mean ± SEM. ⁣^∗^*p* < 0.05 and ⁣^∗∗^*p* < 0.001 vs. Control. ⁣^#^*p* < 0.05 and ⁣^##^*p* < 0.001 vs. HG.

To explore the mechanism of inhibition of fibrosis by Art, the effects of Art on PPAR*γ* activation in HK-2 cells under HG conditions were examined. We found that PPAR*γ* was mainly localized in the cytoplasm (Figure 5a), while a decreased translocation of PPAR*γ* from cytoplasm into nuclei induced by HG was observed (Figure 5b). In addition, Art treatment significantly enhanced the HG-induced PPAR*γ* nuclear translocation (Figure 5c,d, ⁣^∗^*p* < 0.05 and ⁣^∗∗^*p* < 0.001 vs. Control; ⁣^#^*p* < 0.05 and ⁣^##^*p* < 0.001 vs. HG) indicating that Art activated PPAR*γ* expression under HG conditions (Figure 5e). We further confirmed these findings with Western blot and RT-PCR (Figure 6, ⁣^∗^*p* < 0.05 and ⁣^∗∗^*p* < 0.001 vs. Control; ⁣^#^*p* < 0.05 and ⁣^##^*p* < 0.001 vs. HG), which yielded results that were consistent with immunofluorescence staining.

### 4.4. The siRNA Knockdown of PPAR*γ* Reduce the Inhibitory Role of Art in HG-Induced Inflammatory Factors Expression

**Figure 7:**
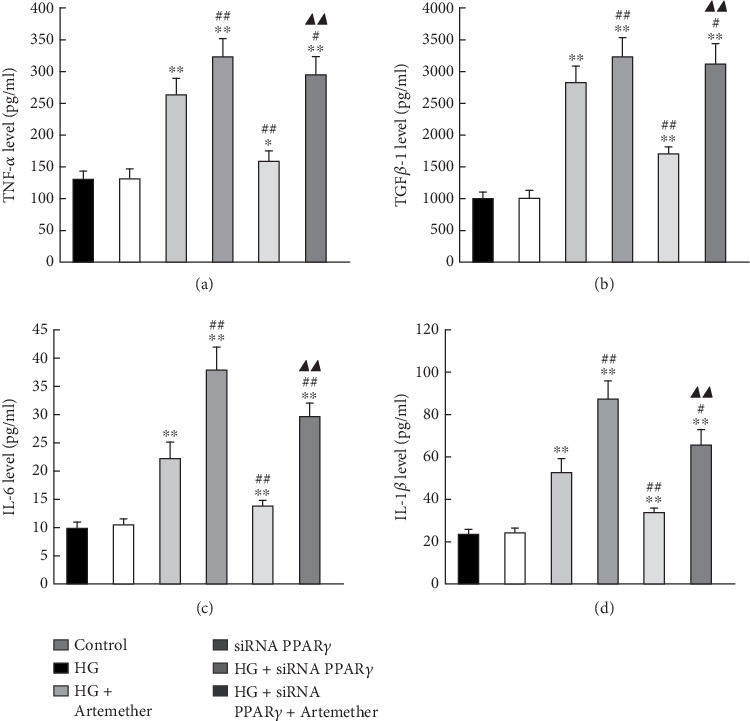
The siRNA knockdown of PPAR*γ* reduce the inhibitory role of Art in HG-induced inflammatory factors expression. siRNA–PPAR*γ* significantly increased HG-induced inflammatory factors levels. (a) TNF-*α*, (b) TGF*β*-1, (c) IL-6, (d) IL-1*β*, while Art treatment decreased their levels (panels a–d). ⁣^∗^*p* < 0.05 and ⁣^∗∗^*p* < 0.001 vs. Control. ⁣^#^*p* < 0.05 and ⁣^##^*p* < 0.001 vs. HG.

To examine the role of PPAR*γ* in the upregulation of inflammatory factors expression, we used PPAR*γ*-siRNA to test the PPAR*γ* real function. The siRNA knockdown of the PPAR*γ* gene was performed and subsequent events of inflammatory factors levels were also examined. Firstly, the siRNA knockdown of the PPAR*γ* protein at various time-points were detected using Western blot analysis after siRNA–PPAR*γ* transfection. The expression level of PPAR*γ* was decreased after siRNA–PPAR*γ* transfection at 24, 48, 72, and 96 h, respectively (Supplemental Figure S1). Secondly, the cells were divided into six groups as following: (1) control group with normal medium; (2) siRNA PPAR*γ* group, subjected to PPAR*γ*–siRNA transfection; (3) HG group, treated with 30 mM HG for 48 h; (4) HG + siRNA PPAR*γ* group, 24 h post-transfection, the cells were treated with 30 mM HG for 48 h; (5) HG + Art group, pretreated with 200 *μ*M Art for 24 h then cells were treated with 30 mM HG for 48 h; (6) HG + siRNA PPAR*γ* + Art group, 24 h post-transfection, the cells were pretreated with 200 *μ*M Art for 24 h then treated with 30 mM HG for 48 h. As shown in Figures 7, 7, 7, and 7, the protein expressions of these inflammatory factor levels (TNF-*α*, TGF*β*-1, IL-6, and IL-1*β*) were upregulated, confirming that HG promotes inflammation in HK-2 cells. Remarkably, pretreatment of the HK-2 cells with Art attenuated the HG-induced upregulation of inflammatory factor levels, which were abolished by siRNA-PPAR*γ*. These data suggest that Art inhibits HG-induced inflammation in HK-2 cells by modulating PPAR*γ* (Figures 7, 7, 7, and 7⁣^∗^*p* < 0.05 and ⁣^∗∗^*p* < 0.001 vs. Control; ⁣^#^*p* < 0.05 and ⁣^##^*p* < 0.001 vs. HG).

### 4.5. PPAR*γ* Is Involved in the Inhibitory Effect of Art on the TGF-*β*/Smad Signaling Pathway

**Figure 8:**
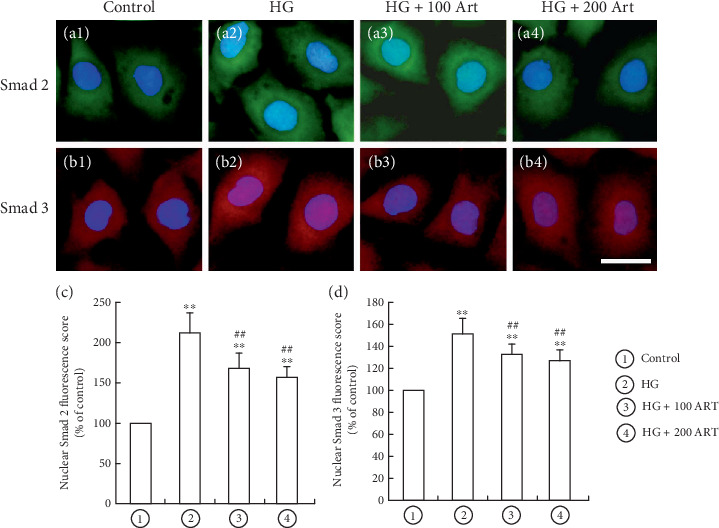
PPAR*γ* is involved in the inhibitory effect of Art on the activation of TGF-*β*/Smad signaling pathway. Representative photo of Samd2 and Samd3 nuclear translocation, assayed by immunofluorescence confocal microscopy in HK-2 cells. a1, b1 the control group; a2, b2, the HG group; a3, b3 the HG + 100 Art group; a4, b4, the HG + 200 Art group. Results are representative of three experiments (bar 10 *μ*m). (c, d) Relative Smad3 and Smad2 fluorescaping score in HK-2 cells. ⁣^∗^*p* < 0.05 and ⁣^∗∗^*p* < 0.001 vs. Control. ⁣^#^*p* < 0.05 and ⁣^##^*p* < 0.001 vs. HG.

**Figure 9:**
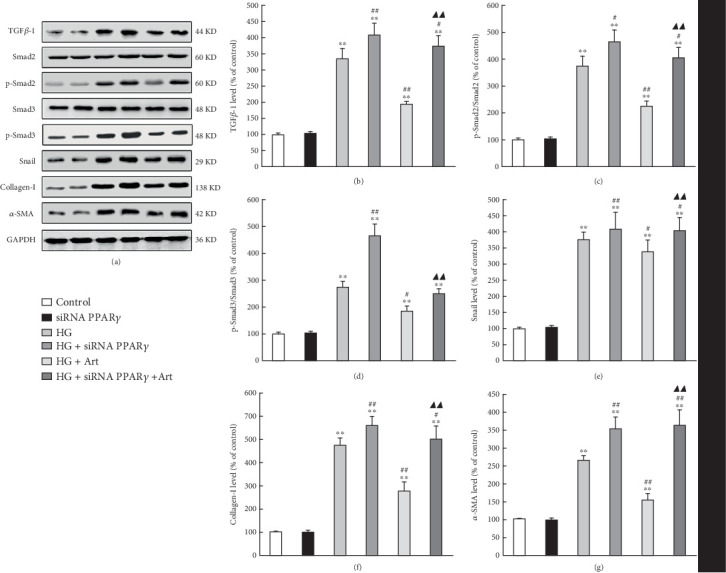
PPAR*γ* is involved in the inhibitory effect of Art on the TGF-*β*/Smad signaling pathway. (a) Silencing PPAR*γ* by siRNA promoted Smad2/3 phosphorylation and fibrosis genes in HK-2 cells stimulated with HG (30 mM, 48 h). Art treatment decreased the Smad2/3 phosphorylation and fibrosis downstream genes under HG stimulation in HK-2 cells. (b–g) Data are expressed as means ± SEM of three independent experiments. ⁣^∗^*p* < 0.05 and ⁣^∗∗^*p* < 0.001 vs. Control. ⁣^#^*p* < 0.05 and ⁣^##^*p* < 0.001 vs. HG. ⁣^▲▲^*p* < 0.001 HG/Art vs. HG/siRNA PPAR-*γ*/Art. GAPDH, glyceraldehyde-3-phosphate dehydrogenase.

TGF-*β*/Smad signaling pathway plays a critical role in the pathogenesis of TIF in DKD. To evaluate the potential impact of Art on the TGF-*β*/Smad signaling pathway, we conducted immunofluorescence staining and Western blot analysis to detect key protein expression when HG stimulated HK-2 cells. Nuclear translocation of Smad complexes is a major step in the TGF-*β*/Smad pathway [29]. Immunofluorescence staining revealed that Art treatment significantly reduced HG-induced Smad2 and Smad3 nuclear translocation (Figures 8a, 8b, 8c, and 8d), ⁣^∗^*p* < 0.05 and ⁣^∗∗^*p* < 0.001 vs. Control; ⁣^#^*p* < 0.05 and ⁣^##^*p* < 0.001 vs. HG). To explore the molecular mechanism behind Art-induced inhibition of Smad2/3 phosphorylation, we knocked down PPAR*γ* in HK-2 cells by siRNA. Real-time PCR confirmed that PPAR*γ* expression in HK-2 cells was effectively suppressed by siRNA. Art treatment was found to substantially decrease HG-induced expression of Smad2 and Smad3 target fibrosis genes including snail, collagen-I, and *α*-SMA. However, knocking down PPAR*γ* enhanced their expression (Figures 9a, 9b, 9c, 9d, 9e, 9f, and 9g, ⁣^∗^*p* < 0.05 and ⁣^∗∗^*p* < 0.001 vs. Control; ⁣^#^*p* < 0.05 and ⁣^##^*p* < 0.001 vs. HG. ⁣^▲▲^*p* < 0.001 HG/Art vs. HG/siRNA PPAR-*γ*/Art). These results suggest that Art negatively controls the TGF-*β*/Smad pathway by PPAR*γ*-mediated inhibition of nuclear translocation and Smad2/3 phosphorylation.

## 5. Discussion

In this study, we demonstrated that Art effectively attenuates HG-induced inflammation and fibrogenesis in human renal tubular epithelial cells by modulating PPAR*γ*-mediated inhibition of the TGF-*β*/Smad signaling pathway. These findings suggest that Art may represent a promising therapeutic candidate for DKD.

Renal fibrosis is a final common pathway in CKD, characterized by excessive accumulation of ECM components such as fibronectin (FN), *α*-SMA, and collagen [3, 5, 30]. Although glomerulosclerosis is a hallmark of DN, the degree of tubulointerstitial injury primarily determines the rate of renal function decline [3–5]. Recent studies increasingly support the antifibrotic effects of artemisinin derivatives. In liver fibrosis, Art induces ferroptosis in HSCs, reducing injury [31, 32]. Art treatment also suppresses fibroblast proliferation, myofibroblast differentiation, collagen deposition, inflammatory cell infiltration, and cytokine expression in renal fibrosis models [33–36]. We employed 100 and 200 *μ*M Art concentrations, which exhibited no toxicity to HK-2 cells in vitro. HG stimulation elevated fibrogenic markers *α*-SMA, collagen-I, and snail in HK-2 cells, which were significantly inhibited by Art pretreatment via modulation of the TGF-*β*/Smad pathway through PPAR*γ* activation. Importantly, the siRNA-mediated PPAR*γ* knockdown abolished Art's antifibrotic effects, underscoring the critical role of PPAR*γ* in mediating its therapeutic action.

Growing evidence indicates that Art and its derivatives possess potent anti-inflammatory and immunoregulatory properties [37]. PPAR*γ* serves as a gatekeeper of ECM and vascular cell homeostasis, playing beneficial roles in renal, cardiac, and pulmonary fibrosis [38]. Previous study indicated that PPAR*γ* activation attenuates lipopolysaccharide-induced inflammation in mouse models, suggesting potential benefits in inflammatory diseases such as chronic obstructive pulmonary disease (COPD) [39]. Art treatment enhances PPAR*γ* activation, reversing cigarette smoke-induced airway injury in bronchial epithelial cells [40]. Broeders et al. suggested that PPARs critically influence DKD pathogenesis via glycemic control and lipid metabolism [41]. Based on these findings, we hypothesized that PPAR*γ* activation contributes to protection against inflammation and fibrosis in diabetes-associated tubulointerstitial injury. Consistent with this, Art treatment reduced TNF-*α*, TGF-*β*1, IL-6, and IL-1*β* levels under HG conditions in HK-2 cells. Conversely, the siRNA-mediated PPAR*γ* knockdown increased inflammatory cytokine expression. Notably, PPAR*γ* was upregulated in response to Art but downregulated under HG exposure. Cotreatment with HG and Art enhanced PPAR*γ* nuclear translocation, suggesting that adaptive upregulation of PPAR*γ*-driven anti-inflammatory mechanisms may be a crucial protective factor in TIF.

It is widely accepted that the TGF-*β*/Smad pathway plays a critical role in the development of TIF in both humans and experimental animals [4, 42]. Moreover, inhibition of TGF-*β*1 expression via PPAR*γ* activation is an effective approach to treating TGF-*β*1–driven fibrosis [43]. Several studies have demonstrated that PPAR*γ* agonists reduce renal interstitial fibrosis and inflammation by suppressing the TGF-*β*1 pathway [44, 45]. Furthermore, the activation of PPAR*γ* has been shown to confer renoprotective effects by modulating the TLR4/TGF-*β*1 axis [46]. Nong et al. reported that Art treatment suppressed the expression of TGF-*β*1 and Smad3, thereby reducing hypertrophic scarring [47]. In the present study, we investigated whether ART's antifibrotic effects against HG-induced fibrogenesis involve the TGF-*β*1 pathway via PPAR*γ* activation in DKD. Our findings revealed that the knockdown of PPAR*γ* using siRNA restored activation of the TGF-*β*/Smad signaling pathway and abrogated the protective effects of Art, which confirm that Art ameliorates HG-induced fibrosis through modulating the PPAR*γ*/TGF-*β*/Smad signaling pathway.

## 6. Conclusions

In DKD, Art attenuates HG-induced fibrosis and inflammation by downregulating fibrogenic markers including Snail, *α*-SMA, and Collagen-I, as well as inflammatory cytokines such as TNF-*α*, TGF-*β*1, IL-6, and IL-1*β*. Furthermore, Art suppresses the TGF-*β*/Smad signaling pathway via PPAR*γ*-dependent inhibition of Smad2/3 phosphorylation and nuclear translocation. This study demonstrates that Art exerts protective effects against HG-induced inflammation and fibrogenesis in HK-2 cells by restraining TGF-*β*/Smad signaling pathway activation, suggesting its potential as a therapeutic strategy for the prevention and treatment of TIF in DKD.

## Data Availability

Data are available if requested.

## Nomenclature

Art: Artemether
ACR: albumin-to-creatinine ratio
AAS: (atomic absorption spectrophotometry)
BUN: urea nitrogen
CKD: chronic kidney disease
PPAR*γ*: proliferator-activated receptor gamma
DKD: diabetic kidney disease
ESKD: end-stage kidney disease
EMT: epithelial-to-mesenchymal transition
ELISA: enzyme-linked immunosorbent assay
FBG: fasting blood glucose
HG: high glucose
IL-1*β*: interleukin-1*β*
IL-6: interleukin 6
PB: phosphate buffer
RPTC: renal proximal tubular cells
STZ: streptozotocin
TGF-*β*1: transforming growth factor *β*1
TNF-*α*: tumor necrosis factor-*α*
TIF: tubulointerstitial fibrosis
sCr: serum creatinine
T2DM: Type 2 diabetes mellitus
UACR: urine albumin-to-creatinine ratio
UUO: unilateral ureteral obstruction

## Supporting Information

Supporting Information Additional supporting information can be found online in the Supporting Information section. Supplemental Figure S1. PPAR*γ* protein and mRNA expression with siRNA knockdown of the PPAR*γ* gene at various time-points. Relative expression of PPAR*γ* was detected using Western blot analysis and RT-PCR at various time-points following siRNA–PPAR*γ* transfection though transient methods. ⁣^∗^*p* < 0.05 and ⁣^∗∗^*p* < 0.001 vs. Control.
