# Supplementary figures and images for: Artemether Attenuates High Glucose-Induced Inflammation and Fibrogenesis in Renal Tubular Epithelial Cells by Modulating the TGF-β/Smad Pathway Via PPARγ Activation
Source: J Diabetes Res. 2025 Nov 29;2025:5052561. doi: 10.1155/jdr/5052561 (PMC12681401; doi:10.1155/jdr/5052561)

## Suppl Fig1

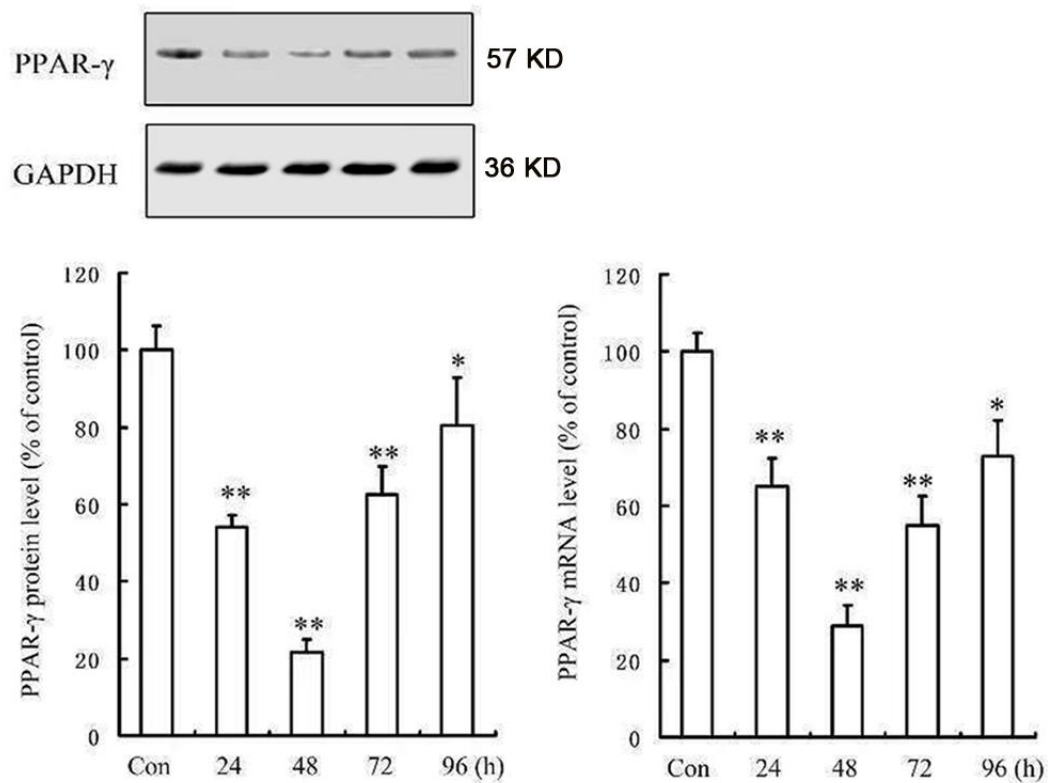

Supplement: Supporting Information — Additional supporting information can be found online in the Supporting Information section. Supplemental Figure S1. PPARγ protein and mRNA expression with siRNA knockdown of the PPARγ gene at various time-points. Relative expression of PPARγ was detected using Western blot analysis and RT-PCR at various time-points following siRNA–PPARγ transfection though transient methods. ⁣∗p < 0.05 and ⁣∗∗p < 0.001 vs. Control. [file 5052561.f1.pdf]
